# Integrating HIV and primary healthcare for key populations: community‐led models from Vietnam, Nigeria and Eswatini

**DOI:** 10.1002/jia2.70027

**Published:** 2025-09-03

**Authors:** Megan Coleman, Christopher Akolo, Acapel Mbanusi, Bhekizitha Sithole, George K. Siberry, Ryan Schowen, Deborah Goldstein

**Affiliations:** ^1^ HIV Technical Advisor for Key Populations Washington DC USA; ^2^ FHI 360 Washington DC USA; ^3^ Key Population Activist Abuja Nigeria; ^4^ FHI 360 Mbabane Eswatini; ^5^ Columbia University Irving Medical Center New York New York USA; ^6^ Technical Advisor HIV Services Washington DC USA

**Keywords:** key populations, HIV, PEPFAR, primary healthcare, integration

## Abstract

**Introduction:**

Key populations (KP), including men who have sex with men, people who inject drugs, sex workers, transgender people and people in closed settings, are disproportionately affected by HIV and face structural and legal barriers to care. While community‐led responses are central to reaching KP, services are often disease‐specific and disconnected from national primary healthcare (PHC) systems. PHC, defined by WHO as a whole‐of‐society approach to delivering integrated and person‐centred services, is rarely designed to meet the broader health needs of KP, who also experience high burdens of non‐communicable diseases, mental health conditions and violence. This paper describes three service delivery models, supported by PEPFAR, that integrate HIV and PHC services for KP in Vietnam, Nigeria and Eswatini.

**Discussion:**

The three models are community‐led, client‐centred, and tailored to KP health and social needs. Each integrates HIV services—including testing, antiretroviral therapy, viral load monitoring, pre‐exposure prophylaxis (PrEP) and advanced HIV disease management—alongside broader PHC services such as mental healthcare, sexual and reproductive health, non‐communicable disease screening and tuberculosis services. All models include structural and community‐based interventions such as gender‐based violence support, stigma reduction, peer navigation and economic empowerment. These services are delivered in safe, trusted spaces by multidisciplinary teams including peer and clinical providers. While the models demonstrate alignment with PHC principles (accessibility, cultural competence, continuity and community empowerment), challenges remain related to integration within national health systems, financing and provider training. Recent U.S. global health policy shifts, including reductions in funding for KP‐specific programming and limited PrEP access, pose additional threats to programme sustainability and client trust.

**Conclusions:**

Integrated models of HIV and PHC for KP can improve access, engagement and health outcomes across a range of services. They represent promising approaches for addressing intersecting health and structural needs, particularly in settings where stigma and criminalization persist. Sustained progress will require inclusion of KP in PHC policies and planning, protection of community‐led services and domestic financing strategies that ensure continuity in the face of shifting donor priorities.

## 1 INTRODUCTION

Global HIV response advocates have historically identified five key populations (KP)—men who have sex with men, people who inject drugs, people in prisons and other closed settings, sex workers and transgender people—but programmes serving these groups are often underfunded, limiting their reach and impact [1]. While KP are at heightened risk of HIV acquisition, they often experience stigmatization, marginalization and discrimination from healthcare providers, as well as punitive laws that limit access to HIV and mainstream health services [2, 3, 4, 5]. KP frequently experience mistreatment, denial of care, stigma and a lack of privacy in health facilities [3, 4, 6, 7, 8].

Historically, donor‐funded HIV services have been vertical and disease‐specific. However, there is a growing shift towards integrating HIV care into national primary healthcare (PHC) systems. Advances in HIV treatment and prevention modalities make them well‐suited for integration; however, this approach is rarely adopted for KP, who often find public facilities unwelcoming or even dangerous [9]. Public facilities may lack clinical skills and competencies for KP; staff may harbour attitudes and biases that discourage KP from accessing care; and unique personal identifiers tracking clients’ healthcare utilization may increase the risk of identification and criminalization.

The World Health Organization (WHO) recommends that KP, like all people, have access to services that meet their broader health needs, services that PHC systems are well‐positioned to provide [10]. In line with WHO definitions, PHC refers to a “whole‐of‐society approach” encompassing integrated health services, multisectoral action and empowered communities. KP often have lower uptake of preventative health services, increasing their risk for non‐communicable diseases (NCDs) [11, 12]. Men who have sex with men experience high rates of mental health disorders, hepatitis B and C, sexually transmitted infections (STIs), and cancers such as anal, liver and Kaposi sarcoma [13, 14]. Female sex workers face elevated maternal morbidity, including abortion complications and pregnancy‐related haemorrhage, along with NCD risk factors such as tobacco and alcohol use, high body mass index (BMI) and physical inactivity [15, 16, 17]. Young KP (ages 10–24) report more unprotected sex, STIs, unintended pregnancy, violence, mental health issues and substance use than both older KP and youth in the general population [18, 19]. Transgender individuals and people who inject drugs also face elevated risks for chronic disease, mental health conditions and infectious complications [14, 20, 21, 22, 23, 24].

Differentiated service delivery, a client‐centred approach adapting services to specific population needs, is one WHO‐recommended model for tailoring HIV services for KP [9]. This approach should equally apply to comprehensive PHC, not just HIV services. Specialized, community‐based clinics—such as KP‐led drop‐in centres and comprehensive one‐stop shops—exemplify this model. While both are typically linked to community‐based organizations, their staffing models differ: one‐stop shops usually include on‐site physicians, while drop‐in centres are often staffed by nurses and trained KP health peers. These clinics often provide co‐located services such as treatment of STIs, mental health support, family planning, tuberculosis (TB) screening, cervical cancer screening, gender‐based violence (GBV) support and substance use services while referring clients for specialized care. They also serve as safe spaces for social interaction, community mobilization, empowerment activities and peer support.

Despite the growing role of these models, limited published evidence exists on how best to integrate HIV and PHC services for KP in low‐ and middle‐income countries. A recent UNAIDS review found that among 114 studies on HIV integration, only 22 reported outcomes specific to KP [25]. Moreover, recent shifts in U.S. global health priorities (such as the closure of USAID and the redirection of PEPFAR resources away from dedicated KP services) further threaten access. Pre‐exposure prophylaxis (PrEP) is now limited to pregnant and breastfeeding women, excluding other high‐risk groups [26]. These changes risk undermining trust, reducing service engagement and reversing progress in epidemic control. To sustain momentum, stakeholders must address stigma, advance equity and support population‐centred models that integrate HIV and PHC services while rebuilding confidence among KP who may feel sidelined.

This paper presents three service delivery models from Vietnam, Nigeria and Eswatini—supported by the U.S. President's Emergency Plan for AIDS Relief (PEPFAR)—that integrate HIV and PHC services for KP. Each example explores programmatic strategies, the role of community leadership, engagement with host governments and approaches to sustainable financing. The goal is to document promising practices and contextual adaptations that may inform the replicability, scale‐up and sustainability of integrated service delivery models for KP in other low‐ and middle‐income settings.

## 2 DISCUSSION

### 2.1 Integrated PHC approaches

PHC, as defined by the WHO, is a whole‐of‐society approach that aims to maximize health and wellbeing through three key components: integrated primary care and essential public health services, multisectoral policy and action, and empowered people and communities [27]. This paper focuses on models of PHC that integrate HIV services with broader health and structural supports tailored to the needs of KP [26].

Facility‐based services at public institutions, as part of differentiated service delivery models, offer access to specialized care and preserve client anonymity when desired. In Vietnam, public (non‐KP‐led) one‐stop shops are frequently affiliated with district hospitals or universities in collaboration with local communities. In contrast, community‐based differentiated service delivery approaches may improve acceptability and accessibility, reducing transport costs, waiting times and stigma that KP may experience at public facilities [10]. Advances in HIV treatment and prevention have facilitated a shift from specialty to primary care, including task shifting to trained community members, themselves often KP members. In Eswatini, urban drop‐in centres are complemented by peer and virtual outreach, case management and mobile clinics targeting KP hotspots (Table 1). In Vietnam, PEPFAR supports KP‐led or KP‐owned stand‐alone one‐stop shops operating primarily in urban areas (Table 1). In Nigeria, KP one‐stop shops introduced in 2015 have expanded with PEPFAR and government support, serving as hubs for community and mobile clinics and extending service availability (Table 1) [28].

**Table 1:** Descriptions of integrated programmes

	Vietnam	Nigeria	Eswatini
**Model**	One‐stop shops typically key population led or owned (stand alone or in public facilities)	Hub and spoke model with central comprehensive clinic (one‐stop shops) connected to community and mobile clinic sites	Community drop‐in centres with mobile and key population outreach at hotspots
**Population served**	Men who have sex with men, transgender persons, partners of key populations	Female sex workers, men who have sex with men, people who inject drugs, transgender persons	Female sex workers, men who have sex with men, people who inject drugs, transgender persons, adolescents, children of key populations, men who purchase sex
**Location**	Mainly urban areas	One‐stop shops are in urban areas with multidisciplinary clinical teams operating at community and mobile outreach sites; home visits	Urban
**Funding**	PEPFAR. Government of Vietnam funds one‐stop shops at public clinics. National social health insurance scheme reimburses one‐stop shops directly for some services. Social enterprise	PEPFAR, Government of Nigeria, private sector; one‐stop shops eligible for state health insurance reimbursement in Lagos State. Social enterprise	PEPFAR, Global Fund to Fight AIDS, Tuberculosis and Malaria
**Integrated PHC services (including HIV)**	Integrated HIV and primary health services including HIV testing, treatment, viral load monitoring, PrEP, advanced HIV disease (AH screening and treatment, alongside mental healthcare, STI management, cervical cancer screening, hypertension and diabetes screening, viral hepatitis care and referrals for advanced NCD care.	Comprehensive PHC including HIV testing, PrEP, Antiretrovial therapy (ART) initiation and refill, viral load monitoring, AHD screening and treatment, NCD care (including hypertension and diabetes), mental health, STI and TB management, radiographic services, malaria and typhoid treatment, and minor illness care, with referral for complex cases.	Integrated services including HIV testing, self‐testing, treatment, viral load monitoring, PrEP, AHD screening, sexual and reproductive health, STI and TB management, mental health support, NCD (diabetes, hypertension, cervical cancer) screening and referrals for advanced care.
**Structural and community‐based interventions**	Counselling on stigma and discrimination, intimate partner violence and gender‐based violence (GBV) services, economic empowerment programmes	Legal services, GBV violence programmes, economic empowerment initiatives and co‐located community centre services for key populations.	Stigma reduction interventions, GBV prevention and response, sensitization and training for public facility staff.
**Staffing**	Multidisciplinary teams including physicians, nurses, pharmacists, lab scientists, counsellors and peer outreach workers. Community partners support outreach, demand generation and client navigation.	PHC teams include medical officers, pharmacists, nurses, lab scientists, adherence counsellors and peer educators; community outreach supported by civil society partners that may be co‐located at the site.	Drop‐in centres staffed by nurses, testing counsellors, psychosocial officers and case managers; mobile teams include nurses, peer navigators and educators.

Bi‐directional referral networks are vital for coordinating care. One‐stop shops utilize established referral processes to link clients to specialized services beyond their scope, similar to general PHC clinics. Vietnam's KP‐led community organizations formalized partnerships with public and private health facilities to address their clients’ needs, additionally providing opportunities for stigma reduction through KP sensitization efforts. In Eswatini, drop‐in centres and mobile community clinics offer some additional non‐HIV clinical services but rely on referral networks to public sector facilities for broader PHC needs.

Stigma and discrimination remain significant barriers to healthcare [10]. Tailored structural interventions addressing broader social determinants of health are essential for programmatic success. Integrated programming in all three countries includes intimate partner and GBV interventions, stigma reduction and economic empowerment initiatives. In Eswatini, KP community members collaborated with the Ministry of Health to develop a training manual for healthcare workers on KP best practices and a standard operating procedure for structural interventions. Best practices include a series of KP‐delivered sensitization trainings and multi‐day stigma and discrimination training for public facilities, taking a whole‐of‐facility approach. Eswatini and Nigeria KP clinics also have onsite or active referral networks to legal/community paralegal services to protect human rights and further reduce stigma and discrimination. The recent inclusion of KP in the work of the Nigerian government's National Human Rights Commission provides an important avenue for addressing human rights violations. In Vietnam, KP‐led organizations actively train on gender sensitivity, stigma and discrimination reduction, and violence prevention while offering virtual interventions addressing stigma. Such structural approaches benefit KP‐focused services and general population facilities where KP may seek PHC or specialty services.

### 2.2 Community engagement

All three programmes actively engage KP community members and civil service organizations in service design and oversight. These models maintain links to, or embed as staff, peer‐led outreach facilitated by trained peer educators or navigators from KP. Community members are integral to outreach, demand generation, communication campaigns, client support and programme monitoring. Strong community engagement is essential to effective PHC integration, ensuring services reflect the needs and priorities of those they serve. PHC services should be customized to the populations they serve rather than to a specific disease programme.

In Vietnam, some one‐stop shops are KP‐led (either donor‐supported or privately owned) and operate in public facilities with designated community engagement spaces. In Nigeria, PEPFAR‐supported one‐stop shops in Lagos partner with community‐based organizations, some of which operate co‐located outreach centres. These partners also support peer‐led demand generation and case management, expanding outside the clinic. Similarly, Eswatini's KP drop‐in centres are supported by active community engagement through their mobile outreach and hot‐spot site activities. To strengthen trusted KP referral networks, the Ministry of Health, KP communities and PEPFAR have collaborated on sensitization efforts and collaborative stigma and discrimination training to increase KP competence at public health facilities.

Community‐led monitoring, an HIV service accountability mechanism, is supported in all three programmes and could readily be expanded to accountability for health services beyond just HIV. These initiatives, led by KP groups or people living with HIV, use client feedback tools such as surveys to improve service quality and uphold the health and human rights of KP [27].

### 2.3 Host government partnerships

Close collaboration with host governments is a critical enabler of the successful implementation of integrated HIV and PHC for KP. Engagement between government stakeholders and KP communities fosters shared accountability, strengthens service delivery and enhances policy implementation. For example, the one‐stop shop model is fully endorsed by Vietnam's Ministry of Health and the Vietnam Administration HIV/AIDS Control programme. The government regulates both public and private sector one‐stop shop clinics, requiring compliance with service‐specific regulations.

In Nigeria, the Ministry of Health established standard operating procedures outlining principles for KP PHC. In Lagos State, the Department of Health has accredited the one‐stop shops as PHC centres, many now empanelled into the state's health insurance programme.

In Eswatini, the Ministry of Health's dedicated KP unit works directly with civil society to co‐develop services. A national KP implementation guide—created in collaboration with community members—outlines the minimum package of services across sectors. Stigma reduction efforts and provider sensitization training have also been implemented collaboratively.

### 2.4 Financing strategies

The PHC programmes for KP in Vietnam, Nigeria and Eswatini use diversified funding models to maintain operational continuity and financial stability. For instance, KP services in Eswatini are primarily supported by PEPFAR and the Global Fund to Fight AIDS, Tuberculosis and Malaria (Table 1).

While donor funding provides essential support for programme adaptation and service expansion, each programme also receives government support, either through direct reimbursement or regulation, aligning services with national health policies and ensuring quality care. In Vietnam, one‐stop shops that were initiated under PEPFAR now receive additional government funding through the national social insurance scheme. In Lagos State, Nigeria, the State Health Insurance scheme provides direct reimbursement to KP‐led clinics for specific services and financed a one‐time infrastructure upgrade to meet regulatory requirements. Additionally, in Nigeria, the Lagos State government subsidizes insurance premiums for indigent KP, strengthening financial risk protection for KP.

Clinic operations are further supported through income generated from KP‐led social enterprises. In Vietnam, community‐based organizations can legally register as businesses under the 2014 Social Enterprise Law. Under this legal framework, KP‐led clinics generate income from direct clinical service delivery (such as fee‐based HIV‐related services, comprehensive private KP‐led clinics or on‐site pharmacies) or ventures such as a transgender‐led health and beauty business initiative and an independent community‐based pharmacy [29]. In Nigeria, some one‐stop shops generate revenue by producing or procuring and reselling HIV prevention products such as condoms and lubricants.

## 3 CONCLUSIONS

Effective healthcare must address an individual's needs while navigating legal and social constraints. This paper presented three PEPFAR‐supported models where KP communities have led PHC services, inclusive of HIV services that included HIV prevention, treatment and broader health supports. These models show potential to enhance access, linkage, retention and stigma reduction among KP. However, sustainability and scalability remain challenges requiring further evaluation and adaptation.

Criminalization, stigma and discrimination can deter individuals from seeking services at public facilities or publicly identified “KP friendly” clinics, particularly when personal identity does not align with programme labelling. Expanding the range of service delivery options allows individuals to access care in environments where they feel safe and respected. Community‐led organizations play a critical role in building trust, linking clients to care and strengthening service quality. Advocacy for improved training, sensitivity and education for all providers, regardless of where they serve patients, is crucial.

KP‐focused health services may continue to rely on external donor support longer than other programmes, especially as overall donor assistance declines and marginalization limits KP participation in policy and planning processes, which in turn affects agenda‐setting and resource allocation. As countries move towards universal health coverage, the failure to include KP in defining and costing benefit packages risks deepening dependence on donor funds—even where domestic resources exist.

Recent shifts in U.S. global health policy—including PEPFAR's decisions to discontinue funding for dedicated KP services and restrict access to PrEP for non‐pregnant populations—further threaten the availability of safe, comprehensive care. These changes may erode trust and reduce service engagement among KPs, particularly in contexts where stigma remains pervasive. To sustain progress in epidemic control, stakeholders must prioritize strategies that rebuild trust, promote equity and ensure continued access to integrated HIV and PHC services for KPs.

Governments and donors can promote integration by involving KP in service design, implementation and oversight; removing policy and funding barriers for community‐based services, and offer flexible service delivery options. Tailored, population‐specific health services (e.g. gender‐affirming care or safe injecting services) should be incorporated into PHC packages. National insurance schemes should reimburse services delivered in community settings, and integration efforts must address structural interventions while leveraging peer outreach. A comprehensive, person‐centred PHC approach is critical to meeting KP health needs and advancing equity. The presented programmes offer valuable lessons for replication, scale‐up and sustainability.

## COMPETING INTERESTS

All authors declare that they have no conflicts of interest to disclose.

## AUTHORS’ CONTRIBUTIONS

MC: Literature search, conceptualization, programme data collection and analysis, writing—original draft. CA: Writing—original draft. AM: Writing—review and editing. RS: Writing—review and editing. GKS: Writing—review and editing. BS: Writing—review and editing. DG: Literature search, conceptualization, writing—original draft. All authors have read and approved the final manuscript.

## DISCLAIMER

The views and opinions in this article are those of the authors and do not necessarily represent the views of USAID, PEPFAR, Credence Management Solutions LLC or the United States Government.

## Data Availability

Data sharing is not applicable to this article as no new datasets were generated or analyzed during the preparation of this commentary. Unpublished programmatic data was shared with the authors to inform the discussion; these data are not publicly available.
